# Suicide and unintentional poisoning mortality trends in the United States, 1987-2006: two unrelated phenomena?

**DOI:** 10.1186/1471-2458-10-705

**Published:** 2010-11-17

**Authors:** Ian RH Rockett, Gerry Hobbs, Diego De Leo, Steven Stack, James L Frost, Alan M Ducatman, Nestor D Kapusta, Rheeda L Walker

**Affiliations:** 1 Department of Community Medicine, West Virginia University, Morgantown, West Virginia, USA; 2 Injury Control Research Center, West Virginia University, Morgantown, West Virginia, USA; 3 Department of Statistics, West Virginia University, Morgantown, West Virginia, USA; 4 Department of Pathology, West Virginia University, Morgantown, West Virginia, USA; 5 Australian Institute for Suicide Research and Prevention, World Health Organization Collaborating Centre for Research and Training in Suicide Prevention, Griffith University, Mt. Gravatt, Queensland, Australia; 6 Department of Criminal Justice and Neuropsychiatry, Wayne State University, Detroit, Michigan, USA; 7 Department of Psychoanalysis and Psychotherapy, Medical University of Vienna, Waehringer Guertel 18-20, A-1090 Vienna, Austria; 8 Department of Psychology, University of Georgia, Athens, Georgia, USA

## Abstract

**Background:**

Two counter trends in injury mortality have been separately reported in the US in recent times - a declining suicide rate and a rapidly rising unintentional poisoning mortality rate. Poisoning suicides are especially difficult to detect, and injury of undetermined intent is the underlying cause-of-death category most likely to reflect this difficulty. We compare suicide and poisoning mortality trends over two decades in a preliminary assessment of their independence and implications for suicide misclassification.

**Methods:**

Description of overall and gender- and age-specific trends using national mortality data from WISQARS, the Web-based Injury Statistics Query and Reporting System, maintained by the Centers for Disease Control and Prevention (CDC). Subjects were the 936,633 residents dying in the 50 states and the District of Columbia between 1987 and 2006 whose underlying cause of death was classified as suicide, unintentional poisoning, or injury mortality of undetermined intent.

**Results:**

The official US suicide rate declined 18% between 1987 and 2000, from 12.71 to 10.43 deaths per 100,000 population. It then increased to 11.15 deaths per 100,000 by 2006, a 7% rise. By contrast to these much smaller rate changes for suicide, the unintentional poisoning mortality rate rose more than fourfold between 1987 and 2006, from 2.19 to 9.22 deaths per 100,000. Only the population aged 65 years and older showed a sustained decline in the suicide rate over the entire observation period. Consistently highest in gender-age comparisons, the elderly male rate declined by 35%. The elderly female rate declined by 43%. Unlike rate trends for the non-elderly, both declines appeared independent of corresponding mortality trends for unintentional poisoning and poisoning of undetermined intent. The elderly also deviated from younger counterparts by having a smaller proportion of their injury deaths of undetermined intent classified as poisoning. Poisoning manifested as a less common method of suicide for this group than other decedents, except for those aged 15-24 years. Although remaining low, the undetermined poisoning mortality rate increased over the observation period.

**Conclusions:**

The official decline in the suicide rate between 1987 and 2000 may have been a partial artifact of misclassification of non-elderly suicides within unintentional poisoning mortality. We recommend in-depth national, regional, and local population-based research investigations of the poisoning-suicide nexus, and endorse calls for widening the scope of the definition of suicide and evaluation of its risk factors.

## Background

US trends in suicide and unintentional poisoning mortality appear to tell quite different stories. The steady decline in the suicide rate between the late 1980 s and 2000 engendered both optimistic discussion and caution concerning attribution to prescriptive use of selective serotonin reuptake inhibitors (SSRIs) and other antidepressants [1-3]. On the other hand, a stronger and more persistent increasing trend in the unintentional poisoning mortality rate has focused attention on the epidemic of fatal prescription and recreational drug overdoses [4-6]. Without compelling corroborative evidence, poisoning suicides are particularly difficult to detect [7,8]. Moreover, a diagnosis or legal ruling of death by suicide is not a default option for medicolegal authorities [9,10], and suicide is highly susceptible to undercounting at local [11,12], state [13], and hence national levels. Indicative of a potentially strong poisoning-suicide nexus, poisoning deaths represent a large component of injury mortality of undetermined intent [14], the category most prone to contain misclassified suicides [15-17].

A recent national multiple-cause-of-death study found that subjects whose mechanism of injury death involved low energy (human or appliance/vehicle), categorized as the less active group, were 46 times more likely to be classified under death of undetermined intent than suicide relative to the more-active group [18]. Alternatively, these two categories could be distinguished as less or more violent. Poisoning mortality predominated in the less active category and suffocation and firearm shooting mortality in the more-active category.

A study of archival data from the New Jersey component of the National Violent Death Reporting System (NVDRS) detected important differences in the degree to which suicide risk factors were reported as present in unintentional and intentional poisoning deaths, and found that a number of risk factors for suicide were more pronounced among the former [19]. Perhaps counterintuitive, substance abuse manifested the largest prevalence differential (90% unintentional versus 24% intentional). However, alcohol and other substance abuse complicates suicide case ascertainment for medical examiners and coroners. While substance use disorders are strong determinants of suicide [20-22], they also diminish the likelihood of a suicide ruling or diagnosis [23-25].

An analysis of data for 13 states from the NVDRS and the National Vital Statistics System inferred that wide variation in classification of poisoning deaths under injury of undetermined intent impaired comparability of suicide and unintentional injury mortality rates [26]. A study of death certificate and medical examiner data for Utah estimated that the unintentional poisoning mortality rate and overall suicide rate were underreported by 61 percent and 10 percent, respectively [27]. A corresponding estimate of underreporting in the poisoning suicide rate was 30%. However, underreporting in both the overall and poisoning suicide rates may have been grossly underestimated by the investigators because they did not allow for possible suicide misclassification under unintentional poisoning. Similarly, underreporting in the unintentional poisoning mortality rate may have been overestimated.

For the period 1987-2006, we question whether US suicide and poisoning mortality rates were unrelated phenomena. In this preliminary evaluation of that question, we first documented the rate trends and then examined corresponding trends for selected gender- and age-specific underlying cause-of-death ratios.

## Methods

We accessed underlying cause-of-death data for the period 1987-2006 from WISQARS, the Web-based Injury Statistics Query and Reporting System, which is maintained by the US Centers for Disease Control and Prevention (CDC) [28]. Causes of death for the period 1987-1998 were precoded under the *International Statistical Classification of Diseases and Related Health Problems, Ninth Revision (ICD-9) *[29], and those for 1999-2006 under the *Tenth Revision (ICD-10) *[30]. Subjects were 936,633 residents of the 50 US states and the District of Columbia whose manner of death during that period was either suicide, operationalized as death from intentional self-harm (ICD-9 E950-E959 or ICD-10 X60.0-X84.9 and Y87.0), or death from injury of undetermined intent (ICD-9 E980-E989 or ICD-10 Y10-Y34 and Y87.2, Y89.9), or death from unintentional poisoning (ICD-9 E850-E869 or ICD-10 X40-X49). For the gender-age comparisons, we excluded decedents under age 15 years at time of death. This group accounted for less than 1% of all official suicides during our observation period.

We calculated three sets of gender- and age-specific underlying cause-of-death ratios. In relating poisoning suicides to all suicides, the first set showed the relative importance of poisoning as a method of suicide in official statistics throughout the observation period. The second set of ratios portrayed the corresponding share of poisoning within injury mortality of undetermined intent, the category most susceptible to suicide misclassification. Thus, this set served as a guide to changing potential for misclassification posed first by undetermined poisoning mortality and then, through inference, by unintentional poisoning mortality. The final set of ratios related unintentional poisoning mortality to suicide.

## Results

**Figure 1:**
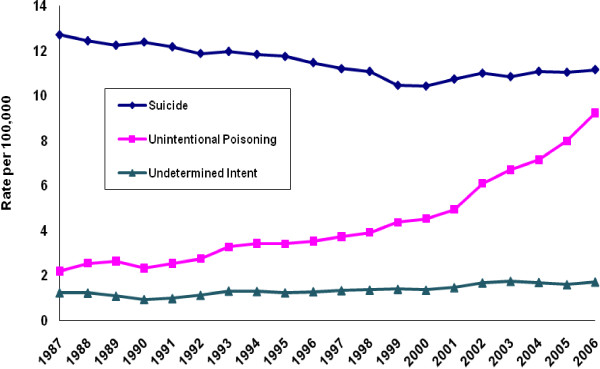
**Mortality rates of suicide, unintentional poisoning, and undetermined intent, United States, 1987-2006**.

**Figure 2:**
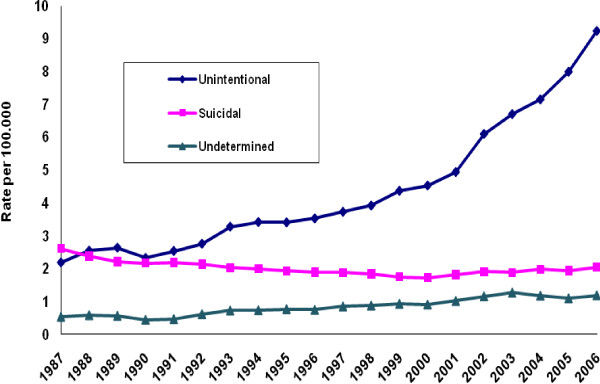
**Poisoning mortality rates by intentionality, United States, 1987-2006**.

Figure 1 depicts trends in US crude mortality rates for suicide, unintentional mortality, and injury of undetermined intent for the period 1987-2006. The suicide rate declined 18% between 1987 and 2000, from 12.71 to 10.43 deaths per 100,000 population. It then increased to 11.15 deaths per 100,000 by 2006, a 7% rise. By contrast, the unintentional poisoning mortality rate increased more than fourfold over our observation period, from 2.19 to 9.22 deaths per 100,000. After rising rather steadily between 1987 and 2000, this rate then accelerated sharply as the suicide rate commenced its more modest increase. Following an early dip, the rate for injury mortality of undetermined intent ascended over most of the observation period, from 1.24 to 1.72 deaths per 100,000. When we distinguished poisoning mortality by intentionality, the suicide poisoning rate showed decline from its high of 2.61 deaths per 100,000 in 1987 (Figure 2). It grossly diverged from the ascending unintentional poisoning mortality rate. The rate of poisoning mortality of undetermined intent progressed upwards, but from a far lower baseline than the rates for both unintentional and suicide poisoning mortality.

While variable across gender and age, the most common suicide methods in the United States in descending order are firearm shooting, poisoning, and suffocation, respectively (data not shown). Collectively, they accounted for 92% of all suicides over the entire observation period, 1987-2006. Between 1987 and 2000, the crude suicide shooting death rate declined from 7.55 to 5.54 per 100,000 population. There was a corresponding rise in the crude suicide suffocation death rate from 1.70 to 2.50. These two changes accounted for a net decline in the suicide rate of 1.21 deaths per 100,000, which represented half of the official suicide rate decline between 1987 and 2000. Crude undetermined intent shooting and suffocation death rates were minuscule, 0.17 and 0.03 per 100,000 in 1987 and 0.12 and 0.04 in 2000, respectively.

**Figure 3:**
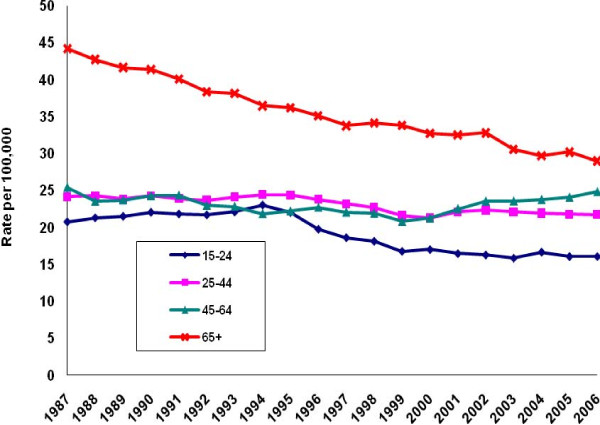
**Male suicide rates by age, United States, 1987-2006**.

**Figure 4:**
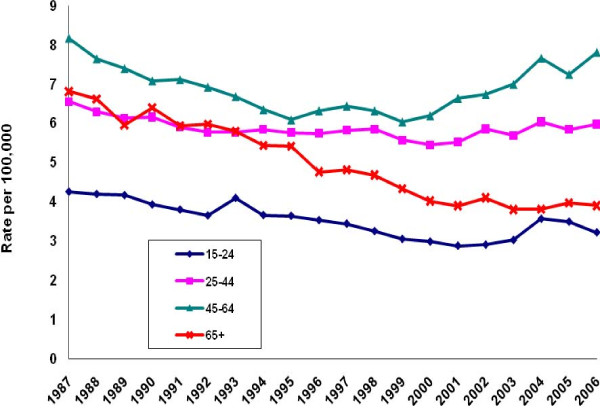
**Female suicide rates by age, United States, 1987-2006**.

Irrespective of gender, the 65-years-and-older age group recorded the only sustained decline in age-specific suicide rates between 1987 and 2006 (Figures 3-4. While the rate for elderly males declined by 35%, it remained the highest by both gender and age. The suicide rate for elderly females declined by 43%. Among females, however, those aged 45-64 recorded the highest suicide rates throughout the observation period.

**Figure 5:**
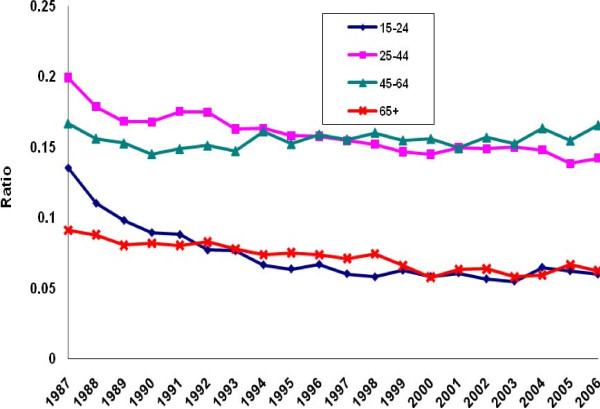
**Ratio of poisoning suicide to all suicide deaths by age, United States males, 1987-2006**.

**Figure 6:**
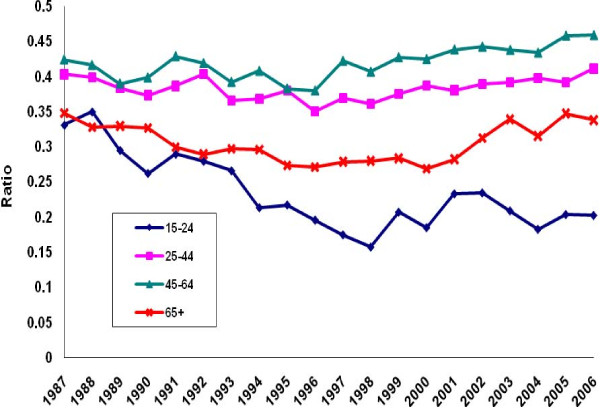
**Ratio of poisoning suicide to all suicide deaths by age, United States females, 1987-2006**.

**Figure 7:**
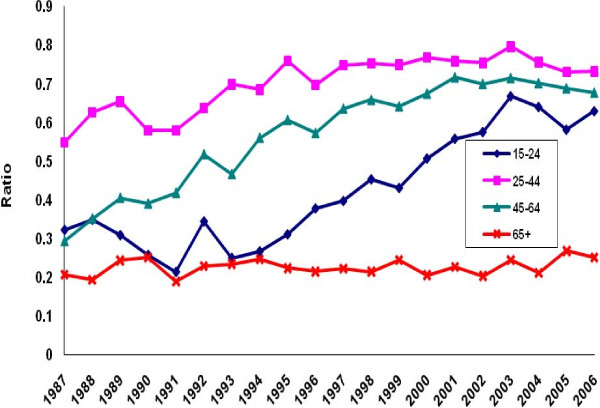
**Ratio of undetermined intent poisoning deaths to all deaths of undetermined intent by age, United States males, 1987-2006**.

**Figure 8:**
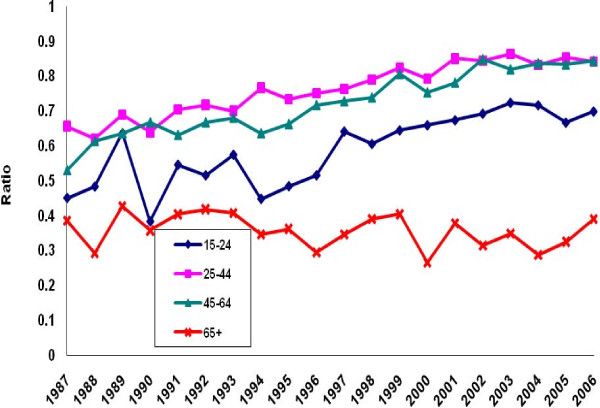
**Ratio of undetermined intent poisoning deaths to all deaths of undetermined intent by age, United States females, 1987-2006**.

**Figure 9:**
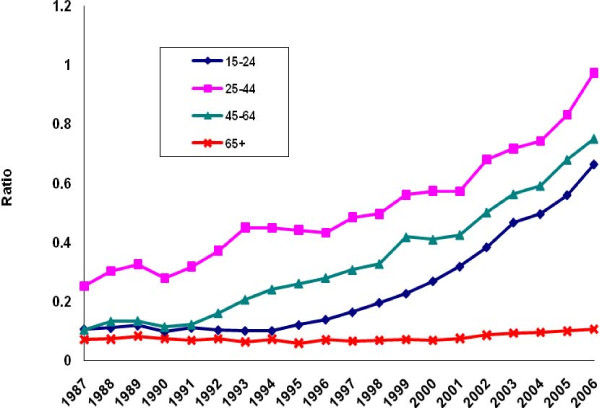
**Ratio of unintentional poisoning to suicide deaths, United States males, 1987-2006**.

**Figure 10:**
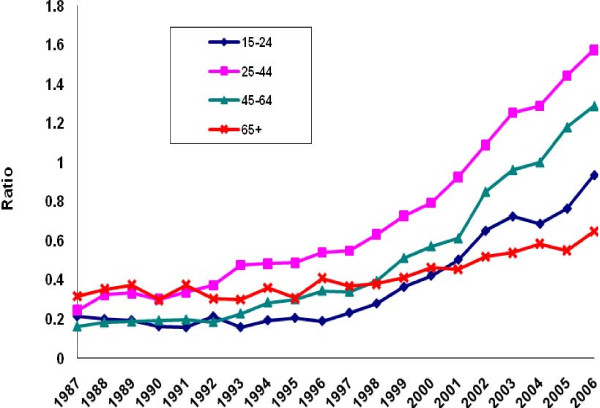
**Ratio of unintentional poisoning to suicide deaths, United States females, 1987-2006**.

There was a perceptible decline in the ratio of poisoning suicide to all suicide mortality among males in each age group except the group aged 45-64 years (Figure 5). Evidence of decline among females was limited to ages 15-24 (Figure 6). Otherwise, the ratio distribution was relatively flat. Poisoning manifested as a less common method of suicide among the youngest and oldest age groups than among the two intermediate groups. For both males and females, the ratio of poisoning mortality of undetermined intent to all injury mortality of undetermined intent tended to rise in all age groups except the oldest (Figures 7-8. Changes were more profound, but the ratio of unintentional poisoning to suicide mortality basically followed suit (Figures 9-10.

## Discussion

A 2002 Institute of Medicine report inferred that suicide etiology and prevention in the United States are complicated by undercounting [31]. Our study yields circumstantial evidence that the official decline in suicide rates between 1987 and 2000 may have been a partial artifact of misclassification within unintentional poisoning mortality. The effect of any such misclassification under poisoning of undetermined intent appeared minimal owing to its very low rates. This study generates additional circumstantial evidence that any underestimation of poisoning suicide, which occurred between 1987 and 2006, primarily involved decedents under age 65 years.

We previously documented that alcohol and other substance abuse can complicate suicide case ascertainment [20-25]. A statewide hospital emergency department study estimated that 27% of patients aged 18 years and older needed substance abuse treatment, where need was assessed by means of self-report and validatory toxicological testing [32]. However, patients aged 65 years and older were only 10-20 percent as likely as younger patients to need such treatment. These comparative findings for hospital emergency department patients, a patently high-risk population [33], strengthen our conclusion that potential poisoning-associated suicide misclassification is least problematic for the elderly. On the other hand, younger decedents appear much more likely than older counterparts to undergo toxicological testing, as we infer from their far higher national autopsy rates [34]. However, indirect evidence indicates that medicolegal authorities exercise more caution in ruling or diagnosing suicide among younger than older decedents [18].

There are numerous social contributors to suicide underreporting in general and specific underreporting of poisoning suicide. A New York City report in the 1980 s indicated that scarce resources and personnel and policy changes influenced medical examiners to classify many suicides as unintentional injury deaths [12], a possible artifactual contributor to the contemporaneous and precipitous decline in the suicide rate. While we cannot identify the independent contributions of underfunding of cause-of-death investigations, changing policy, or the prevailing epidemic of unintentional poisoning deaths, our findings indicate that such forces collectively impede suicide case ascertainment, probably explain some of the data trends, and may indirectly foster unwarranted complacency about the suicide burden.

Case ascertainment and rate changes for suicide in the United States can be compared unfavorably to homicide and fatal unintentional motor vehicle traffic trauma. Suicide is more susceptible to underenumeration than these other leading causes of injury mortality because of social pressure and marked contrasts in resources for the affected agencies from medical examiners and coroners to the police, judiciary, and various public and private ancillary organizations. For example, indicating greater fastidiousness in homicide versus suicide investigations, a federal report showed that 92% of homicides in 2003 were autopsied versus 55% of suicides, 77% of undetermined intent deaths, and 73% of unintentional poisoning deaths [34]. Mean annual age-adjusted death rates for 1987-1989 and 2004-2006, based on the US 2000 standard population, show declines of 28% for homicide and 22% for fatal motor vehicular traffic trauma [28]. By contrast, the suicide rate decreased 13% and the unintentional poisoning mortality rate increased 233%. Corresponding changes for poisoning of undetermined intent and poisoning suicide were a 102% rise and a 21% decline. At 8 per 100,000, mean crude and age-adjusted unintentional poisoning mortality rates surpassed corresponding homicide rates by one-third in the 2004-2006 triennium. Given the great magnitude and substantial growth of poisoning deaths, we recommend that their investigations be appropriately resourced so that decedent intentionality can be comprehensively assessed together with type and dose of toxin.

Our inferential data, in concert with our justified concern about potential adverse implications for suicide misclassification from stressed resources, challenge the official record that poisoning became a less common method of suicide during an epidemic of unintentional poisoning mortality and era of unprecedented consumer access to a growing pharmacy of potentially lethal toxins [35]. Access to lethal methods, including prescription drugs, affects suicide rates [36]. Documenting a sharp rise in fatal poisonings between 1999 and 2006, a new federal government report showed a marked increase in the proportion involving opioid analgesics relative to illicit drugs like heroin and cocaine [37]. Methadone was the leading cause of death among the opioids, but other significant killers included oxycodone and hydrocodone. There was also a high prevalence of concomitant dual or multiple drug use. Related to physician prescription of stronger analgesics for pain management, the increase in opioid deaths coincided with increased sales for each drug type, including methadone [38]. Moreover, the increase in methadone deaths has been more closely associated with pharmaceutical sales than with activity in narcotics treatment programs. Media reporting might also be implicated in the epidemic of opioid mortality [39], and prescription drug diversion is becoming a core issue [38,40]. While demographic data lack drug specificity, the largest increase in the poisoning mortality rate occurred at ages 50-59 years, followed by ages 15-29 years [5]. Distinguishing gender, the highest rate increases were registered for females ages 50-59, followed by females ages 20-29 and males ages 15-19 and 50-59, respectively. Together with measurement or estimation of dosage, identification of specific drugs and combinations of drugs is crucial for developing effective prevention strategies. Deficits in this information likely adversely impact medicolegal assessment of intentionality.

Complicating evaluation of decedent intent, and etiologic understanding of suicide, are the competing beneficial and harmful exposures which characterize the rapid rise of psychotropic medication [41]. With controversial benefit for youth and the youngest adults, prescribed use of SSRIs alone, for example, seems neutral on suicidal behavior and protects against suicidal ideation in adults ages 25-64 [42]. This use diminishes the risk of both suicidality and suicidal behavior in those aged 65 years and older. Consideration of our results, in conjunction with those from systematic reviews of antidepressants and suicide risk [43,44], leads us to recommend a gender- and age-specific evaluation of the association between psychopharmacology and the decline in elderly suicide rates in particular. It would be prudent for such a study to factor in autopsy rates [45], since they vary with age [34].

This exploratory research possesses a number of limitations. We only indirectly addressed our question concerning the independence of observed trends in suicide and unintentional poisoning mortality rates. Analysis was confined to population-level, underlying cause-of-death data based on death certificates. In precluding poisoning comorbidity, these data underestimate the role of toxic substances in injury mortality, irrespective of manner of death. Moreover, there is no national medical examiner and coroner database that would permit us to analyze and examine the evidence that medicolegal authorities compile and utilize to ascertain suicide. In addition, suicide typically occurs in a local context, whose heterogeneous constellation of determinants includes geography, climate, living and working conditions, access to means of suicide, community attitudes towards suicide and cooperation with death investigators, as well as psychiatric, familial, religious, cultural, and employment variables, race/ethnicity, lifestyles and risk behaviors, and other decedent characteristics.

We think unlikely, but acknowledge in light of our research limitations, that observed trends in national suicide and unintentional poisoning mortality rates could be independent. The clear trend in overall poisoning mortality lends weight to a new argument that suicide prevention must address the gamut of risky behaviors inducing self-destruction beyond those clearly implicating deliberate intent [26,46], and another that the definition of suicide needs broadening [47]. Interviews with survivors of near fatal "unintentional" overdoses documented an ambivalent attitude towards potential death at time of overdose [48,49]. Such a finding implied a life-threatening or suicidal component in their self-poisoning. Suicide may be a failed or failing category for classifying and preventing self-harm in the United States [50,51], owing to presumed difficulties confronting many medicolegal authorities in evaluating intent during soaring caseloads from the burgeoning poisoning epidemic. Prescription and nonprescription drugs comprise the vanguard of substances with high potential for lethality and abuse. Our results reinforce an identified need for the National Violent Death Reporting System to incorporate unintentional poisonings and other unintentional injury deaths which implicate self-harm, irrespective of decedent intent [26].

## Conclusions

The official decline in the suicide rate between 1987 and 2000 may have been a partial artifact of misclassification of non-elderly suicides within unintentional poisoning mortality. From a public health perspective, our study advances the cause of evidence-based suicide research and prevention [52] by raising the possibility that suicide misclassification is a growing problem in a highly developed nation. It contributes clinically by presenting data which call for specific analysis of the relationship between psychopharmacology and the sustained decline in elderly suicide rates.

Our findings reveal an imperative for in-depth national, regional, and local population-based investigations of the poisoning-suicide nexus. Optimally, such research would combine death certificate, medical examiner, coroner, emergency response, and law enforcement data, augmented by psychological autopsies and community-based surveys and ethnographic studies of suicide. We anticipate that implementation of such a comprehensive research agenda would yield transformational knowledge about suicide, while recognizing that it would trigger formidable, but not intractable, ethical concerns for investigators and their scientific review boards. More specifically, we predict that the new knowledge would generate radical improvements in suicide surveillance; risk group delineation; risk factor identification; interventions and evaluation; policy; and prevention, particularly regarding the role of ethical drugs. At the other extreme, adherence to the status quo only ensures that suicide remains underestimated as a national public health problem.

## Competing interests

The authors declare that they have no competing interests.

## Authors' contributions

IRHR conceived and designed the study. IRHR obtained, prepared, and managed the data, and IRH and GH performed the analyses. IRHR, SS, and NDK conducted the literature review. IRHR, GH, DD, SS, JLF, AMD, NDK, and RLW interpreted the findings, drafted the manuscript, and read and approved the final version.

## Pre-publication history

The pre-publication history for this paper can be accessed here:

http://www.biomedcentral.com/1471-2458/10/705/prepub
